# Association between height and hypercholesterolemia in adults: a nationwide population-based study in Korea

**DOI:** 10.1186/s12944-019-1148-7

**Published:** 2019-11-15

**Authors:** Mi Yeon Lee, Ga Eun Nam, Kyungdo Han, Da Hye Kim, Yang Hyun Kim, Kyung Hwan Cho, Yong Gyu Park

**Affiliations:** 1 Department of Biomedicine & Health Science, Graduate School, The Catholic University of Korea, 222 Banpo-daero, Seocho-gu, Seoul, 06591 Republic of Korea; 2 Division of Biostatistics, Department of R&D Management, Kangbuk Samsung Hospital, Sungkyunkwan University School of Medicine, 29 Saemunan-ro, Jongno-gu, Seoul, 03181 Republic of Korea; 3 Department of Family Medicine, Korea University Anam Hospital, Korea University College of Medicine, 73 Goryeodae-ro, Seongbuk-gu, Seoul, 02841 Republic of Korea; 4 Department of Medical Lifescience, College of Medicine, The Catholic University of Korea, 222 Banpo-daero, Seocho-gu, Seoul, 06591 Republic of Korea

**Keywords:** Adults, Height, Hypercholesterolemia, Total cholesterol

## Abstract

**Background:**

Previous studies reported that stature is inversely related to the risk of cardiovascular disease. However, there is limited evidence on the association between height and lipid profiles. We aimed to examine the association of height with total cholesterol and hypercholesterolemia based on the nationally representative dataset of Korean adults.

**Methods:**

The data of 13,701 adults aged ≥19 years who participated in the Korea National Health and Nutrition Examination Survey (2013–2015) were used in this nationwide population-based cross-sectional study. Hypercholesterolemia was defined as a serum total cholesterol level ≥ 240 mg/dL or use of lipid-lowering medications. Multivariable linear regression and logistic regression analyses were used to examine the association of height with mean total cholesterol level and odds ratios (ORs) of hypercholesterolemia.

**Results:**

Approximately 17% of participants had hypercholesterolemia. Mean total cholesterol levels decreased in the higher quartile (Q) groups of height after adjusting for confounding variables including age, sex, body mass index, smoking status, alcohol consumption, physical activity, income, educational level, hypertension, and diabetes mellitus (P for trend = 0.035). After adjusting for these potential confounding variables, the adjusted ORs of hypercholesterolemia were significantly lower in the Q3 and Q4 groups than in the Q1 group; ORs decreased in the higher quartile groups of height (OR: 0.83, 95% confidence interval: 0.71–0.99 in Q3; 0.81, 0.69–0.95 in Q4, P for trend = 0.007). The association between height (Q4 vs. Q1–Q3) and hypercholesterolemia was stronger in men or individuals without hypertension or diabetes than in women or individuals with such diseases.

**Conclusions:**

Height is inversely associated with total cholesterol level and odds of hypercholesterolemia among Korean adults. Childhood environment related to short stature may be associated with hypercholesterolemia and cardiovascular health in adulthood.

## Background

Cardiovascular disease (CVD) is the leading cause of mortality and imposes a huge burden on patients and society worldwide [1]. Elevated total cholesterol level has been recognized as one of most important risk factors for CVD. It is a key component of cardiovascular risk prediction models that are widely used in clinical practice to estimate cardiovascular risk and to determine the appropriate clinical treatment [2–4]. Genetics and lifestyles including unhealthy dietary habits, physical inactivity, and obesity have been known to cause dyslipidemia, which is characterized by increased levels of total cholesterol, triglycerides, and low-density lipoprotein cholesterol (LDL-C) and decreased levels of high-density lipoprotein cholesterol (HDL-C) [5]. In addition, previous studies support the possible association between height and dyslipidemia.

Height is an easily measured anthropometric parameter that is usually determined during childhood and adolescence by genetic predisposition, nutrition, exercise, and social factors [6]. Several previous epidemiologic studies have reported an inverse association between height and CVDs such as coronary heart disease and stroke [7–16]. Although the mechanisms underlying this association are unclear, since both height and CVD are associated with social position and genetic factors, the combination of these factors possibly leads to this association [17, 18]. In addition, poor nutritional status during childhood, which results in suboptimal growth, may be associated with increased risk of coronary heart disease in later life [19, 20]. These findings enabled us to hypothesize that abnormal lipid profile, as a major risk factor of CVD, may be associated with short stature. However, cardiovascular risk factors are influenced by complex events that occur during childhood and adolescence and during adulthood. Moreover, only a limited number of studies evaluating the association between height and lipid profiles have been conducted [21, 22]. Therefore, we aimed to examine the association of height with total cholesterol levels and hypercholesterolemia based on the nationwide population-based dataset of South Korean adults.

## Materials and methods

### Data source and study participants

This nationwide population-based cross-sectional study was based on data from the Korea National Health and Nutrition Examination Survey (KNHANES) conducted between 2013 and 2015. The KNHANES has been managed by the Korea Centers for Disease Control and Prevention and the Korean Ministry of Health and Welfare since 1998 to monitor health and nutritional status and to estimate the health statistics of the South Korean population. The KNHANES uses a complex, stratified, and multistep probability sample design with proportional allocation based on age, sex, and geographic area from the 2005 National Census Registry to obtain a representative sample of the civilian, non-institutionalized South Korean population. This survey consists of a health interview survey, a health examination survey, and a nutritional survey. Data from these surveys provide a variety of information regarding sociodemographics, health behaviors, health status, and laboratory tests. Detailed information regarding KNHANES has been previously published [23].

In this study, we initially included individuals who participated in the 2013–2015 KNHANES and excluded individuals aged < 19 years (*n* = 4914) and those who had missing variables (*n* = 4333). Finally, data from 13,701 individuals (6801 men and 6900 women) were analyzed. All participants provided a written informed consent. This study adhered to the ethical principles for medical research involving human subjects of the Helsinki Declaration. The survey was approved by the institutional review board of Korea Centers for Disease Control and Prevention (number: 2013-07CON-03-4C in 2013; 2013-12EXP-03-5C in 2014; 2015-01-02-6C in 2015).

### Assessment and definitions

Participants’ lifestyle and sociodemographic data were collected using a self-reported questionnaire. Individuals were categorized as nonsmokers and current smokers based on their smoking history. Alcohol drinkers were defined as individuals who consumed ≥1 glass of alcohol per month within the last year. We categorized physical activity based on frequency, duration, and type of exercise; individuals who performed walking exercise ≥30 min ≥5 days per week or resistance exercise ≥2 days per week were defined as regular exercisers. Income level was divided into quartile groups of household income level, and the lowest quartile group was defined as the low-income group. Educational level was divided into two groups: ≤middle school graduate and ≥ high school dropout. Anthropometric measurements were performed by trained staff following the standard procedure.

Height and waist circumference were measured to the nearest 0.1 cm, while body weight was measured to the nearest 0.1 kg, while participants wore light clothing without shoes. Body mass index (BMI) was calculated as body weight (kg) divided by height in meters (m) squared. Blood pressure was measured using a standard mercury sphygmomanometer with the participant in a sitting position after at least 5 min of rest. Blood samples were obtained after overnight fasting. Serum levels of glucose, total cholesterol, triglycerides, HDL-C, and LDL-C were measured with an enzymatic method using a Hitachi Automatic Analyzer 7600 (Hitachi, Tokyo, Japan). High total cholesterol (hypercholesterolemia) was defined as a serum total cholesterol level of ≥240 mg/dL or use of lipid-lowering medications [24]. Hypertension was defined as systolic and diastolic blood pressure of ≥140/90 mmHg or use of antihypertensive medications. Diabetes mellitus (DM) was defined as a serum fasting glucose level of ≥126 mg/dL, as diagnosed by a physician, and the use of antidiabetic medications.

### Statistical analyses

SAS version 9.4 (SAS Institute Inc., Cary, NC, USA) was used for statistical analysis. The SAS survey procedure was adopted to analyze complex survey data, taking into account the complex sampling design, and to provide nationally representative prevalence estimates. Statistical weights were assigned to each participant to produce results that can represent the entire Korean population. The characteristics of the study participants were presented as mean ± standard error or percentage (standard error) according to the presence of hypercholesterolemia. The values were compared using the independent t-test for continuous variables and chi-square test for categorical variables. Multivariable linear regression analysis was performed to obtain multivariable adjusted means of total cholesterol according to the quartile (Q) groups of height. Multivariable logistic regression analysis was performed by calculating the odds ratios (ORs) and 95% confidence intervals (CIs) of hypercholesterolemia according to the quartile groups of height with the lowest quartile (Q1) group of height as the reference group. In each analysis, model 1 was unadjusted, while model 2 was adjusted for age and sex. Model 3 was further adjusted for BMI, smoking status, alcohol consumption, physical activity, income, educational level, hypertension, and DM. Subgroup analysis was also performed to evaluate the association between height (Q4 vs. Q1–Q3) and hypercholesterolemia in subgroups stratified by age, sex, smoking status, BMI, hypertension, and DM. A two-tailed *P*-value of < 0.05 was considered significant.

## Results

### Characteristics of study participants

Table 1 presents the characteristics of study participants with and without hypercholesterolemia. There were 2324 (17%) individuals who had hypercholesterolemia. The mean age was higher, and the proportion of men was lower, in individuals with hypercholesterolemia than in those without hypercholesterolemia. The proportions of current smoker, alcohol drinker, and regular exerciser were significantly higher in individuals without hypercholesterolemia than in those with hypercholesterolemia. Socioeconomic status such as income and educational level, was significantly lower in the hypercholesterolemia group than in the non-hypercholesterolemia group. Mean values of height were 161.5 ± 0.3 cm and 165.0 ± 0.1 cm in individuals with and without hypercholesterolemia, respectively. Mean levels of cardiometabolic parameters such as weight, BMI, waist circumference, blood pressure, fasting glucose, total cholesterol, triglycerides, and LDL-C were significantly higher in individuals with hypercholesterolemia than in those without hypercholesterolemia. The prevalence of hypertension and DM was also higher in the hypercholesterolemia group than in the non-hypercholesterolemia group. Approximately 45.5% of individuals with hypercholesterolemia were treated with lipid-lowering medications.

**Table 1:** Characteristics of study participants

Hypercholesterolemia			
	No	Yes	*P*-value^a^
N	11,377	2324	
Age (years)	43.7 ± 0.2	54.8 ± 0.4	< 0.001
Sex (male)	50.5 (0.5)	44.5 (1.1)	< 0.001
Current smoker	22.9 (0.5)	19.8 (1.1)	0.009
Alcohol drinker	60.9 (0.6)	51.4 (1.2)	< 0.001
Regular exerciser	50.8 (0.6)	44.4 (1.3)	< 0.001
Income (the lowest quartile)	12.6 (0.5)	20.3 (1.1)	< 0.001
Education (≤middle school graduate)	21.2 (0.6)	40.8 (1.3)	< 0.001
Height (cm)	165.0 ± 0.1	161.5 ± 0.3	< 0.001
Weight (kg)	64.4 ± 0.1	65.5 ± 0.3	0.001
BMI (kg/m^2^)	23.5 ± 0.0	25.0 ± 0.1	< 0.001
Waist circumference (cm)	80.5 ± 0.1	85.0 ± 0.2	< 0.001
Systolic blood pressure (mmHg)	115.2 ± 0.2	122.7 ± 0.4	< 0.001
Diastolic blood pressure (mmHg)	74.8 ± 0.2	77.4 ± 0.3	< 0.001
Fasting glucose (mg/dL)	97.1 ± 0.2	106.4 ± 0.7	< 0.001
Total cholesterol (mg/dL)	183.0 ± 0.3	219.6 ± 1.4	< 0.001
Triglycerides (mg/dL)^b^	104.9 (103.5–106.4)	149.1 (144.7–153.6)	< 0.001
HDL-C (mg/dL)	51.1 ± 0.1	51.9 ± 0.3	0.016
LDL-C (mg/dL)	106.3 ± 0.3	130.5 ± 1.3	< 0.001
Hypertension	20.3 (0.5)	45.3 (1.3)	< 0.001
Diabetes mellitus	7.0 (0.3)	21.3 (1.0)	< 0.001
Lipid-lowering medication	.	45.5 (1.3)	

Data were presented as mean ± standard error or percentage (standard error)

^a^ *P*-values were obtained using an independent t-test for continuous variables and a chi-square test for categorical variables

^b^ Log transformation was performed for the analysis and presented as geometric mean (95% confidence interval)

### Adjusted mean levels of total cholesterol according to the quartile groups of height.

Table 2 shows the mean levels of total cholesterol according to the quartile groups of height. The values significantly decreased in the higher quartile groups of height in the unadjusted model (model 1, P for trend = 0.032). This association persisted even after adjusting for potential confounding variables including age, sex, BMI, smoking status, alcohol consumption, physical activity income, education, hypertension, and DM (model 3, P for trend = 0.035).

**Table 2:** Adjusted mean values of total cholesterol according to the quartile groups of height

Total cholesterol (mg/dL)			
Quartiles of height	Model 1^a^	Model 2^b^	Model 3^c^
Q1	188.80 ± 0.71	190.22 ± 0.70	190.41 ± 0.68
Q2	188.96 ± 0.72	190.43 ± 0.71	190.11 ± 0.70
Q3	188.40 ± 0.70	189.93 ± 0.67	189.75 ± 0.66
Q4	186.74 ± 0.76	188.51 ± 0.73	188.38 ± 0.73
P for trend	0.032	0.066	0.035

Data were presented as mean ± standard error

Values were obtained using multivariable linear regression analysis

^a^ Model 1 was unadjusted

^b^ Model 2 was adjusted for age and sex

^c^ Model 3 was adjusted for age, sex, body mass index, smoking status, alcohol consumption, physical activity, income, education, hypertension, and diabetes mellitus

### Adjusted ORs (95% CIs) of hypercholesterolemia according to the categories of height

**Fig. 1:**
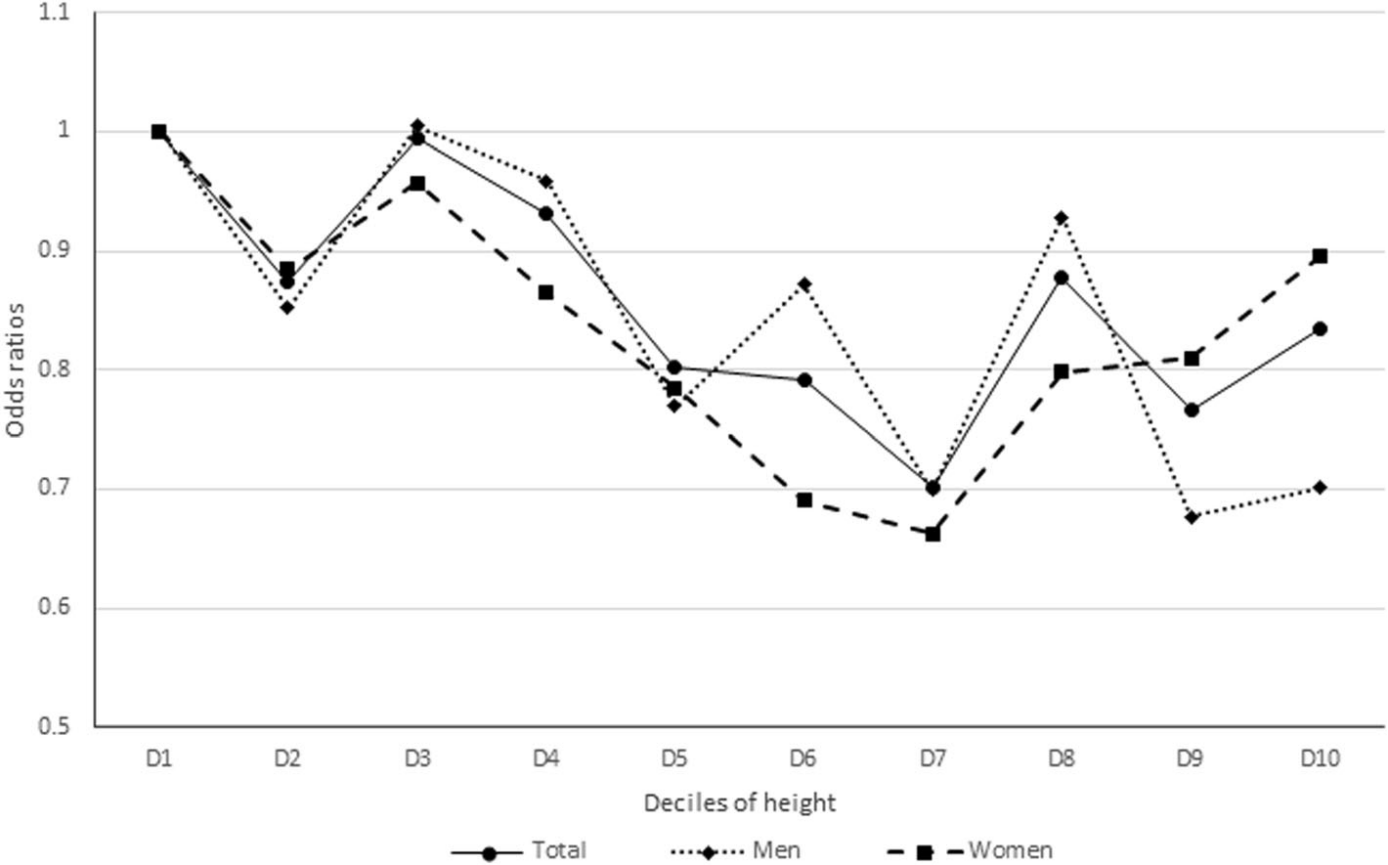
Adjusted odds ratios of hypercholesterolemia in decile groups of height compared with the lowest decile group (P for trend = 0.020 in total participants, 0.028 in men, and 0.099 in women)

Table 3 shows the adjusted ORs and 95% CIs of hypercholesterolemia in the second highest quartile (Q2), the third highest quartile (Q3), and the highest quartile (Q4) groups of height compared to those in the lowest quartile group of height (Q1). The odds of hypercholesterolemia was significantly lower in the Q3 and Q4 groups than in the Q1 group, and the odds showed significantly decreasing trends in the higher quartile groups of height. These associations were still observed even after adjusting for potential confounding variables (model 3, OR 0.91, 95% CI: 0.78–1.06 in Q2; 0.83, 0.71–0.99 in Q3; 0.81, 0.69–0.95 in Q4; P for trend = 0.007). In addition, Fig. 1 shows the ORs and 95% CIs of hypercholesterolemia in the decile groups of height compared to the lowest decile group of height (D1). ORs significantly decreased in higher decile groups among total participants and men (P for trend = 0.020 in total participants, 0.028 in men, and 0.099 in women).

**Table 3:** Odds ratios (95% confidence intervals) of hypercholesterolemia according to quartile groups of height

Quartiles of height	Model 1^a^	Model 2^b^	Model 3^c^
Q1	1 (ref.)	1 (ref.)	1 (ref.)
Q2	0.92 (0.80–1.07)	0.92 (0.79–1.08)	0.91 (0.78–1.06)
Q3	0.83 (0.71–0.96)	0.83 (0.71–0.98)	0.83 (0.71–0.99)
Q4	0.78 (0.67–0.91)	0.81 (0.69–0.95)	0.81 (0.69–0.95)
P for Trend	0.001	0.006	0.007

Values were obtained using multivariable logistic regression analysis

^a^ Model 1 was unadjusted

^b^ Model 2 was adjusted for age and sex

^c^ Model 3 was adjusted for age, sex, body mass index, smoking status, alcohol consumption, physical activity, income, education, hypertension, and diabetes mellitus

### Subgroup analysis

Table 4 shows the results of subgroup analyses stratified by age, sex, smoking status, BMI, hypertension, and DM regarding the association between height and hypercholesterolemia. Compared with that in the Q1–Q3 groups, ORs for hypercholesterolemia in the Q4 group of height were not significantly different among subgroups stratified by age, smoking status, and BMI (P for interaction = 0.101, 0.064, and 0.079). The association had significant interactions with sex, hypertension, and DM (P for interaction = 0.019, 0.044, and 0.040) and was stronger in men or individuals without hypertension or DM than in women or individuals with hypertension or DM.

**Table 4:** Subgroup analysis

Subgroup	OR (95% CI)	P for interaction
Age (years)		0.101
19–39	0.62 (0.41–0.95)	
40–64	0.87 (0.74–1.03)	
≥65	1.09 (0.88–1.36)	
Sex		0.019
Male	0.75 (0.62–0.92)	
Female	1.00 (0.85–1.19)	
Smoking status		0.064
Nonsmoker	0.88 (0.76–1.01)	
Current smoker	0.94 (0.69–1.26)	
Body mass index (kg/m^2^)		0.079
< 25	0.87 (0.75–1.02)	
≥25	0.91 (0.74–1.13)	
Hypertension		0.044
No	0.84 (0.71–0.996)	
Yes	0.97 (0.79–1.19)	
Diabetes mellitus		0.040
No	0.87 (0.76–1.00)	
Yes	1.01 (0.75–1.37)	

Values were obtained using multivariable logistic regression analysis after adjusting for age, sex, body mass index, smoking status, alcohol consumption, physical activity, income, education, hypertension, and diabetes mellitus

## Discussion

In this study, mean total cholesterol levels and odds of hypercholesterolemia (total cholesterol ≥240 mg/dL or treatment with lipid-lowering medication) significantly decreased in taller individuals. We found that height is inversely related to total cholesterol level and hypercholesterolemia, independent of other classical cardiovascular risk factors in Korean adults. In addition, these associations were stronger in men and individuals without hypertension or DM. Our findings suggest that short stature may be an associated factor of increased total cholesterol level in adults. Additionally, factors related with short stature in childhood and adolescence may influence the lipid profiles of individuals when they reach adulthood, which can lead to future CVD.

There has been very limited evidence on the association between height and lipid profiles. A hospital-based cross-sectional study of Japanese men aged 30–59 years reported that height was associated with dyslipidemia only in non-obese individuals; the adjusted OR for dyslipidemia was 0.90 (95% CI: 0.82–0.99) for an increment of one standard deviation in height [21]. A cross-sectional study of 2338 men and women in the UK reported an inverse relationship between height and age-adjusted total cholesterol level [22]. Our findings are consistent with the reports of previous studies and considered a broader span of age and confounders. Therefore, our findings seem to provide additional important epidemiologic evidence of the inverse association between height and total cholesterol level. In addition, recent previous studies reported the association of height with CVDs and mortality. A 41.3-year follow-up study of young and socially homogeneous men in the US reported that height was inversely associated with mortalities from CVD and coronary heart disease [13]. A study of population-based twin cohorts in Denmark, Finland, and Sweden reported that height was inversely associated with coronary heart disease mortality in monozygotic discordant twins, suggesting the direct influence of environmental factors on this relationship [14]. A Korean prospective study of middle-aged men found an opposing association of height with all-cause mortality and mortality from hemorrhagic stroke, but not with cardiovascular mortality [15]. Studies based on the Asia Pacific Cohort Studies Collaboration database reported an inverse relationship between height and CVD [16]. Most studies on height and health outcomes determine the inverse association between height and cardiovascular health, closely related with blood lipid level, and support our findings regarding the association between height and hypercholesterolemia.

The underlying mechanisms linking height and total cholesterol level seem to be unclear. Since both shorter stature and increased cardiovascular risk are partly determined by genetics, this may explain the link between height and hypercholesterolemia in our study. The findings from the aforementioned studies, which indicated that the associations of height with dyslipidemia and stroke were significant only in individuals without obesity, may support this point. Obese people may be more influenced by current circumstances including unhealthy lifestyle than non-obese people [25]. Childhood and adulthood socioeconomic conditions may play a role in the inverse association between height and hypercholesterolemia. Poor socioeconomic status during childhood or adulthood is associated with adult stature, while current adiposity is closely related to hypercholesterolemia [26]. Although hypercholesterolemia was found to be associated with low current socioeconomic status, we could not assess the childhood socioeconomic status of the participants. Hence, further studies are needed to clarify the role of socioeconomic status in the association between height and hypercholesterolemia. Height was positively correlated with bone marrow activity in studies that revealed inverse associations of short height with high white blood cell counts and anemia. Therefore, decreased bone marrow activity in people with short stature may induce unfavorable lipid profiles because bone is important in the regulation of glucose and lipid metabolism [27–29].

Interestingly, the inverse association between height and high total cholesterol was stronger in men and individuals without hypertension or DM. The aforementioned study, which examined the association between height and dyslipidemia in men, supports this association. Furthermore, several previous studies evaluating the association between height and cardiovascular disease mostly reported a more significant association in men than in women. The significant findings reported in individuals without hypertension or DM seemed to be in line with the findings of previous studies examining the association between height and dyslipidemia among non-obese people. They suggest the possible effect of childhood environment on health. However, we did not find any significant interaction of BMI with the association between height and hypercholesterolemia. Hence, further studies are warranted to determine the role of childhood condition in the occurrence of dyslipidemia in adulthood.

Our study has several limitations. First, due to the cross-sectional design of the study, we could not confirm the causal relationship between height and serum total cholesterol. Second, we have focused on total cholesterol, and therefore did not include other lipid-related parameters such as triglycerides, HDL-C and LDL-C. Further studies are required to assess other lipid profiles. Third, total cholesterol was measured only once; the measurement obtained may be inaccurate or influenced by several factors. Fourth, we only considered one ethnic group; therefore, it may be difficult to apply our findings to other races or ethnicities. Despite these limitations, the major strength of our study was that it covered a large, nationally representative dataset of the South Korean population. We were able to consider various confounding variables including socioeconomic status, health behaviors, and current health status as well as perform stratified analyses according to various factors. In addition, to the best of our knowledge, this was the first Korean study to evaluate the relationship between height and total cholesterol.

## Conclusions

From this nationally representative study of South Korean adults, we found an inverse association between height and serum total cholesterol and hypercholesterolemia independent of socioeconomic, lifestyle, and health status including obesity, hypertension, and DM, which are related to high total cholesterol levels. Short stature may be a factor related to hypercholesterolemia, which is a risk factor of future cardiovascular disease. Childhood environment related to stature may be associated with hypercholesterolemia and cardiovascular health in adulthood.

## Data Availability

The dataset is publicly available on the Korea Centers for Disease Control and Prevention website.

## Abbreviations

BMI: Body mass index
CI: Confidence interval
CVD: Cardiovascular disease
DM: Diabetes mellitus
HDL-C: High-density lipoprotein cholesterol
KNHANES: Korea National Health and Nutrition Examination Survey
LDL-C: Low-density lipoprotein cholesterol
OR: Odds ratio
Q: Quartile

## References

[1] Benjamin EJ, Blaha MJ, Chiuve SE, Cushman M, Das SR, Deo R (2017). Heart disease and stroke Statistics-2017 update: a report from the American Heart Association. Circulation.

[2] Ray KK, Kastelein JJ, Boekholdt SM, Nicholls SJ, Khaw KT, Ballantyne CM (2014). The ACC/AHA 2013 guideline on the treatment of blood cholesterol to reduce atherosclerotic cardiovascular disease risk in adults: the good the bad and the uncertain: a comparison with ESC/EAS guidelines for the management of dyslipidaemias 2011. Eur Heart J.

[3] Peters SA, Singhateh Y, Mackay D, Huxley RR, Woodward M (2016). Total cholesterol as a risk factor for coronary heart disease and stroke in women compared with men: a systematic review and meta-analysis. Atherosclerosis..

[4] Lewington S, Whitlock G, Clarke R, Sherliker P, Emberson J, Prospective Studies Collaboration (2007). Blood cholesterol and vascular mortality by age, sex, and blood pressure: a meta-analysis of individual data from 61 prospective studies with 55,000 vascular deaths. Lancet.

[5] Kushner PA, Cobble ME (2016). Hypertriglyceridemia: the importance of identifying patients at risk. Postgrad Med.

[6] Gunnell D (2002). Can adult anthropometry be used as a 'biomarker' for prenatal and childhood exposures?. Int J Epidemiol.

[7] Yarnell JW, Limb ES, Layzell JM, Baker IA (1992). Height: a risk marker for ischaemic heart disease: prospective results from the Caerphilly and speedwell heart disease studies. Eur Heart J.

[8] Cook NR, Hebert PR, Satterfield S, Taylor JO, Buring JE, Hennekens CH (1994). Height, lung function, and mortality from cardiovascular disease among the elderly. Am J Epidemiol.

[9] Kannam JP, Levy D, Larson M, Wilson PW (1994). Short stature and risk for mortality and cardiovascular disease events. The Framingham Heart Study. Circulation.

[10] Rich-Edwards JW, Manson JE, Stampfer MJ, Colditz GA, Willett WC, Rosner B (1995). Height and the risk of cardiovascular disease in women. Am J Epidemiol.

[11] Wannamethee SG, Shaper AG, Whincup PH, Walker M (1998). Adult height, stroke, and coronary heart disease. Am J Epidemiol.

[12] Jousilahti P, Tuomilehto J, Vartiainen E, Eriksson J, Puska P (2000). Relation of adult height to cause-specific and total mortality: a prospective follow-up study of 31,199 middle-aged men and women in Finland. Am J Epidemiol.

[13] McCarron P, Okasha M, McEwen J, Smith GD (2002). Height in young adulthood and risk of death from cardiorespiratory disease: a prospective study of male former students of Glasgow University, Scotland. Am J Epidemiol.

[14] Silventoinen K, Zdravkovic S, Skytthe A, McCarron P, Herskind AM, Koskenvuo M (2006). Association between height and coronary heart disease mortality: a prospective study of 35,000 twin pairs. Am J Epidemiol.

[15] Song YM, Smith GD, Sung J (2003). Adult height and cause-specific mortality: a large prospective study of south Korean men. Am J Epidemiol.

[16] Lee CM, Barzi F, Woodward M, Batty GD, Giles GG, Wong JW (2009). Adult height and the risks of cardiovascular disease and major causes of death in the Asia-Pacific region: 21,000 deaths in 510,000 men and women. Int J Epidemiol.

[17] Silventoinen K, Lahelma E, Lundberg O, Rahkonen O (2001). Body height, birth cohort and social background in Finland and Sweden. Eur J Pub Health.

[18] Kaplan GA, Keil JE (1993). Socioeconomic factors and cardiovascular disease: a review of the literature. Circulation.

[19] Kusin JA, Kardjati S, Houtkooper JM, Renqvist UH (1992). Energy supplementation during pregnancy and postnatal growth. Lancet..

[20] Barker DJ (1995). The fetal and infant origins of disease. Eur J Clin Investig.

[21] Shimizu Y, Yoshimine H, Nagayoshi M, Kadota K, Takahashi K, Izumino K (2016). Height correlates with dyslipidemia in non-overweight middle-aged Japanese men. J Physiol Anthropol.

[22] Gunnell D, Whitley E, Upton MN, McConnachie A, Smith GD, Watt GC (2003). Associations of height, leg length, and lung function with cardiovascular risk factors in the Midspan family study. J Epidemiol Community Health.

[23] Kweon S, Kim Y, Jang MJ, Kim Y, Kim K, Choi S (2014). Data resource profile: the Korea National Health and nutrition examination survey (KNHANES). Int J Epidemiol.

[24] National Cholesterol Education Program (NCEP) Expert Panel on Detection, Evaluation and Treatment of High Blood Cholesterol in Adults (Adult Treatment Panel III) (2002). Third Report of the National Cholesterol Education Program (NCEP) Expert Panel on Detection, Evaluation, and Treatment of High Blood Cholesterol in Adults (Adult Treatment Panel III) final report. Circulation.

[25] Nelson CP, Hamby SE, Saleheen D, Hopewell JC, Zeng L, Assimes TL (2015). Genetically determined height and coronary artery disease. N Engl J Med.

[26] Isasi CR, Jung M, Parrinello CM, Kaplan RC, Kim R, Crespo NC (2016). Association of Childhood Economic Hardship with adult height and adult adiposity among Hispanics/Latinos. The HCHS/SOL Socio-Cultural Ancillary Study. PLoS One.

[27] Shimizu Y, Yoshimine H, Nagayoshi M, Kadota K, Takahashi K, Izumino K (2016). Short stature is an inflammatory disadvantage among middle-aged Japanese men. Environ Health Prev Med.

[28] Shimizu Y, Nakazato M, Sekita T, Kadota K, Miura Y, Arima K (2015). Height and drinking status in relation to risk of anemia in rural adult healthy Japanese men: the Nagasaki Islands study. Aging Male.

[29] Sato M, Asada N, Kawano Y, Wakahashi K, Minagawa K, Kawano H (2013). Osteocytes regulate primary lymphoid organs and fat metabolism. Cell Metab.

